# Characterization of Sheep Wool as a Sustainable Material for Acoustic Applications

**DOI:** 10.3390/ma10111277

**Published:** 2017-11-07

**Authors:** Romina del Rey, Antonio Uris, Jesús Alba, Pilar Candelas

**Affiliations:** 1 Centro de Tecnologías Físicas, Universitat Politècnica de València, EPS Gandia, C/Paraninf, 1, 46730 Grao de Gandia, Valencia, Spain; jesalba@fis.upv.es; 2 Centro de Tecnologías Físicas, Universitat Politècnica de Valencia, Camino de Vera s/n, 46022 Valencia, Spain; auris@fis.upv.es (A.U.); pcandelas@fis.upv.es (P.C.)

**Keywords:** natural material, sound absorption, flow resistance, sheep wool, delany-bazley model

## Abstract

In recent years, natural materials are becoming a valid alternative to traditional sound absorbers due to reduced production costs and environmental protection. This paper reports the acoustical characterization of sheep wool. Measurements on normal incidence and diffuse-incidence sound absorption coefficients of different samples are reported. The airflow resistance has also been measured. The results prove that sheep wool has a comparable sound absorption performance to that of mineral wool or recycled polyurethane foam. An empirical model is used to calculate the sound absorption of sheep wool samples. A reasonable agreement on the acoustic absorption of all sheep wool samples is obtained.

## 1. Introduction

In conventional construction, most of the materials used respect little or do not respect at all the environment, since they require high energy expenditure for their extraction, transport, and transformation. In addition, they incorporate chemicals that can be detrimental to human health into materials to improve their technical characteristics. At present, the demand for a more sustainable construction has gone from being a matter of personal choice to being a regulated sector in order to implement measures that improve the environmental behavior of infrastructures and buildings. Construction activity is a great consumer of natural resources. Buildings continue to be a direct cause of pollution after they have been built due to their emissions and their impact on territory. Sustainable construction takes into account the consumption of resources, the environmental impact it produces, and the specific risks to human health. Thus, ecological materials must be natural, renewable, and durable, and they must have a low environmental impact for their manufacture, placement, and maintenance.

In recent years, the development of new renewable materials has focused the attention of the research community. The origin of these materials can be vegetable or animal so their manufacture has a low environmental impact due to the energy saved in the production process. Several papers focused their investigations on thermal insulation [1,2,3,4,5,6,7,8]. Other authors have studied natural fibers for acoustic applications [9,10,11,12,13,14,15,16,17].

This paper describes the work carried out as part of the BIAEFIREMAT project to develop a new eco-materials and sustainable constructive solutions based on the use of waste and renewable raw materials. It is based on the fact that sheep wool is an excellent natural material, with very good characteristics for thermal insulation, moisture management, and sound absorption. However, due to their natural origin, the homogeneity of the fibers is not controlled and each hair or wool fiber can come from different sheep, breeds, or skin areas. It is intended to convert residues of sheep wool into new materials with acoustic applications both in architectural and environmental acoustics. The sheepskin generates well-known wool fibers that are used to make natural materials. These materials could be substitutes of others more aggressive with the environment.

The aim of this paper is to examine the airflow resistance and sound absorption coefficient of a natural and renewable material such as sheep wool as an alternative to classical sound absorbers. The sound absorption capabilities of the sheep wool presented in this paper were measured in an impedance tube and in a reverberation chamber. The sound absorption coefficient has also been calculated by using an empirical model.

## 2. Recall of the Sheep Wool Fiber Characteristics and the Manufacturing Process

**Figure 1:**
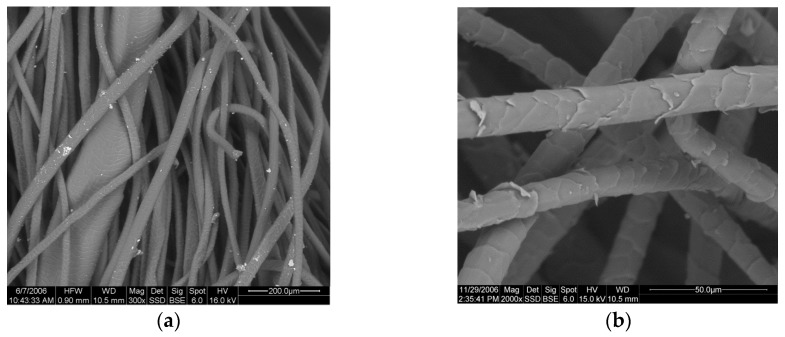
Electronic microscope details of sheep wool fibers. (**a**) 200 μm rank; (**b**) 50 μm rank.

Sheep wool comes in the form of a corrugated fiber having a diameter of 16 to 40 μm and a total length of 35–350 mm. Figure 1 shows electronic microscope details of sheep wool fibers. A sheep wool fiber of 16 μm would have a size similar to mineral fibers. A 33–36 μm sheep wool fiber would be roughly the same size as PET polyester fibers (33 μm) [18] or Kenaf fibers (36 μm) [19]. Unlike synthetic fibers, sheep fibers do not have a fixed thickness. Their thickness range has a standard deviation of 2 μm, according to the consulted scientific works [20]. The fiber diameter also depends on the breed of the sheep. Merina is the predominant breed in Spain. In the Merina and related breeds, the fleece is closed and the wool has no brightness and presents variable uniformity: 60–80 mm length, 18–20 μm fineness, and ripple of 100 mm. The wash performance after shearing to remove natural impurities such as remains of vegetation, urine, and above all grease was 38–42%. It is considered a fine fiber. A priori, it would be comparable to mineral fibers such as fiberglass, and studies of this type of wool should focus on the same applications. This is the best quality sheep wool.

Another type of ovine breed is known as Entrefino group. These are of wide geographic diffusion and of varied productive orientation. When first bred, their main objective was wool production, but today, production has been diversified with meat-wool or meat-milk-wool exploitations. These animals have a semi-closed medium-size fleece. The wool is characterized by its varying brightness, variable uniformity, 70–80 mm length, 28–30 μm fineness, ripple of 40–60 mm, and a thorough wash performance of 42–48%. These fibers would be comparable with polyester fibers (PET) [18].

Finally there is another sheep breed called Churro. The Churro breed is characterized by wool that is shiny, has very low uniformity, 80–120 mm length, 35–40 μm fineness, a low ripple and a thorough wash performance of 46–50%. These fibers would be comparable with other natural fibers such as Kenaf fibers (36 μm) [19]. This is the second best quality sheep wool. 

The manufacturing process of sheep wool is the classic of a nonwoven. It is carried out via dry and thermo-fusion. Sheep wool fibers are “combed” and introduced into the machinery, where they are mixed with polyester fibers obtained from recycled PET flakes. The caula ends up generating sheep wool by the thermo-fusion of the PET fiber that acts as a binder (the fiber melts at 140–150 °C). The material comes out compressed to the desired density depending on the capacity of the machinery. It is a process similar to the manufacture of conventional PET [18]. The difference is that the final product has 80% sheep wool fiber (first quality, second quality, or blend), and the remaining 20% is PET fiber. An important issue is that the machinery used for these new fibers is the same as that used in the textile sector for non-woven materials. The machine should be cleaned only if fibers are changed. 

## 3. Acoustic Measurement Set-Up

### 3.1. Airflow Resistance

**Figure 2:**
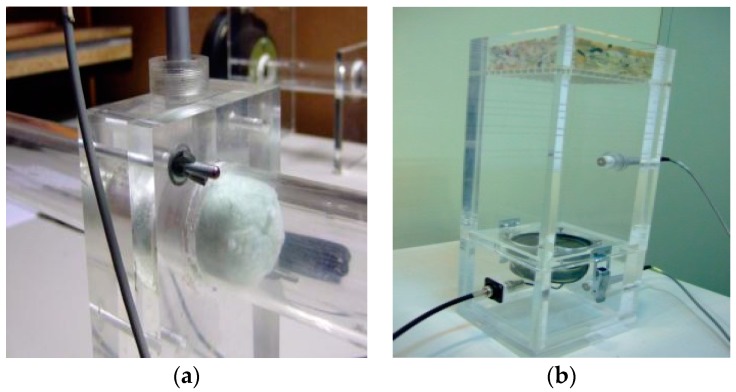
Airflow measurement systems. (**a**) Ingard & Dear’s indirect method; (**b**) Dragonetti et al. method.

To evaluate the airflow resistance of sheep wool samples, two indirect measurement methods were used: Ingard & Dear's indirect method [21] and the Dragonetti et al. method [22]. Both of them allow us to obtain the specific airflow resistance of sound absorbing materials. Ingard & Dear’s method is based on measurements with an impedance tube and two microphones. The Dragonetti et al. method is based on measurement through cavities with different volumes. Details of each of these two measurement methods, similarities and differences between them and the normalized procedure can be found in [23]. Figure 2 shows both airflow measurement systems. 

### 3.2. Sound Absorption Coefficient at Normal Incidence

The sound absorption coefficient at normal incidence, *α*, is the quotient between the acoustic energy absorbed by the surface of the test sample and the incident acoustic energy, for a plane acoustic wave at normal incidence. ISO 10534-2 standard [24] establishes a test procedure to determine the sound absorption coefficient for normal incidence of acoustic absorbers by means of an impedance tube, two microphone positions, and a digital analysis system signal. The measurements described in the standard procedure are useful in basic research and product development.

### 3.3. Sound Absorption Coefficient Measurement in Reverberation Chamber

**Figure 3:**
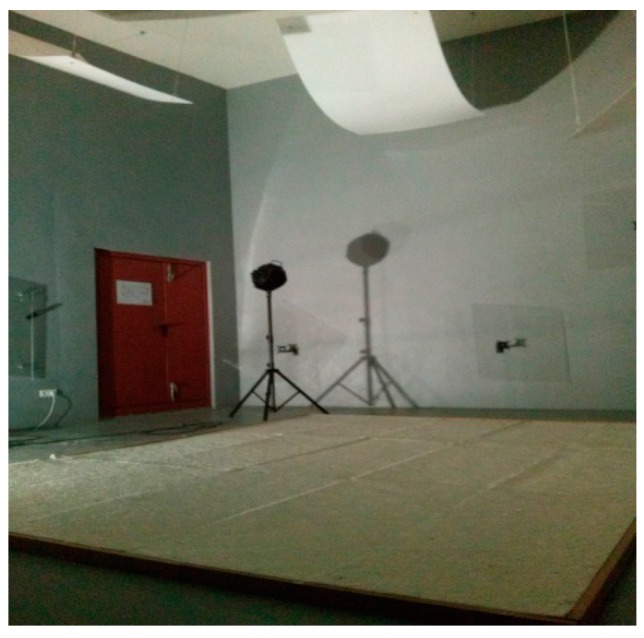
Normalized reverberation chamber at the EPS Gandia at the Universitat Politécnica de València.

ISO 354 standard [25] establishes the measurement procedure of the sound absorption coefficient in a diffuse field. This coefficient is obtained from measurements of the reverberation time, with and without sample, inside a reverberation chamber. Measurements were carried out in the normalized reverberation chamber at the EPS Gandia at the Universitat Politécnica de València (EPSG-UPV). This measurement method requires large samples. Each sample size under investigation had 12 m^2^. Figure 3 shows an image of the normalized reverberation chamber at the EPS Gandia at the Universitat Politécnica de València. 

ISO 11654 standard [26] establishes a procedure for obtaining a single parameter, the weighted sound absorption coefficient, *α*_w_, to evaluate the degree of sound absorption provided by the material. This weighted value is obtained from the 1/3 octave band sound absorption coefficient values measured in the reverberation chamber. The weighted sound absorption coefficient allows to rate the absorbent material as indicated in Table 1.

### 3.4. Tested Samples

In the study reported here, seven sheep wool samples with different compositions were used. The composition of all samples included 20% PET and a variable percentage of wool of first and second quality. Table 2 shows the composition of each considered sample, its density, weight, and thickness, and the code used for each sample.

## 4. Results and Discussion

Three replicates of each sample were prepared and measured so that three values of flow resistance for each sample were obtained. The largest and smallest values are taken as the interval if the typical deviation was lower than 2%. Table 3 presents the results of airflow resistance for the seven samples considered. By comparing different samples results, it is found that there are no great variations on airflow resistance. This is due to the fact that there is a close relationship between airflow resistance, density, and fiber diameter [9]. In this case, there are no large variations in density or fiber diameter, so the airflow resistance values have little variation between samples. The differences are caused by the material heterogeneity, which increases the dispersion of the results [23].

**Figure 4:**
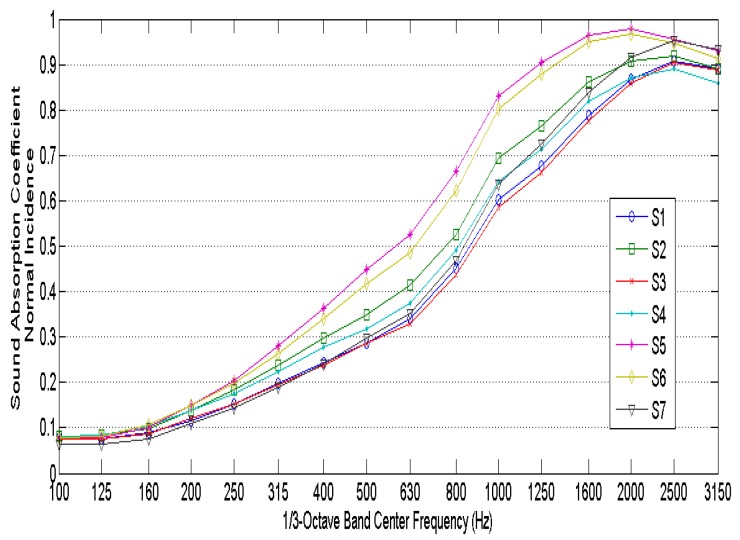
Measured normal incidence sound absorption coefficients.

Figure 4 shows the measured normal incidence sound absorption coefficients of the seven samples considered. The results show high sound absorption coefficient values at mid and high frequencies, making it an excellent sound absorbing material. It is observed that the higher sound absorption coefficients are obtained for the higher thicknesses.

**Figure 5:**
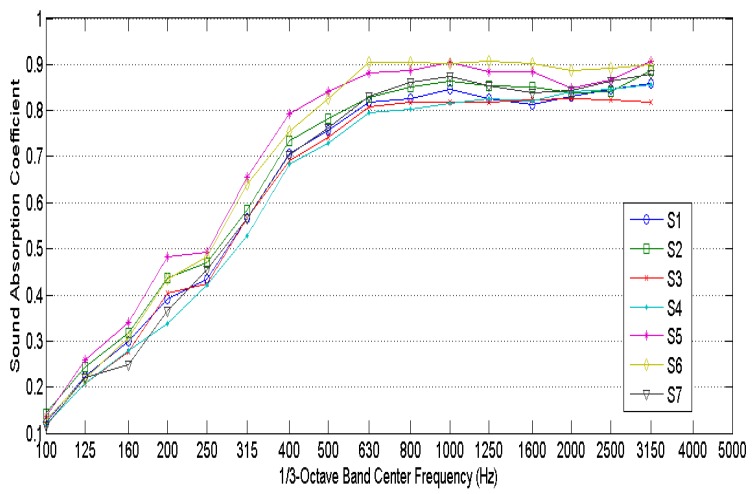
Measured diffuse field sound absorption coefficients.

**Figure 6:**
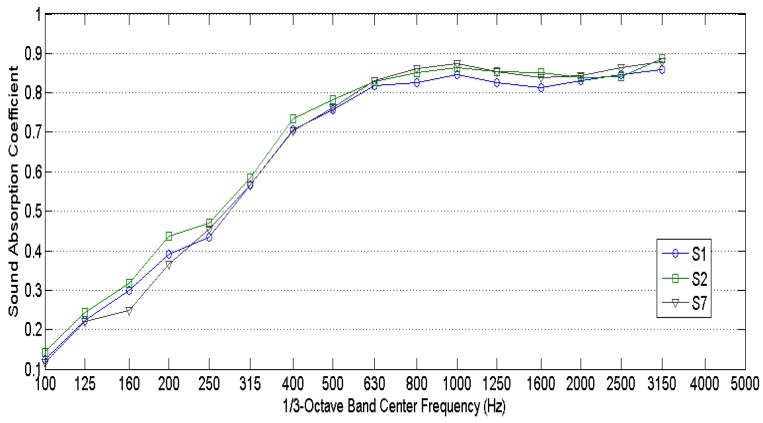
Measured diffuse field sound absorption coefficients of samples S1, S2, and S7.

For room acoustics and environmental applications, normal incidence sound absorption is not directly usable. It is more practical and more representative of the performance of the material to use diffuse-field sound absorption. The measured diffuse field sound absorption coefficients are shown in Figure 5. As expected, the diffuse field sound absorption coefficient values are higher than the normal incidence sound values for the same samples. Figure 5 reveals that there are no great variations between sound absorption properties of measured samples due to the fact that differences between densities and thicknesses in all measured samples are relatively small: samples densities range between 25–40 kg/m^3^ while sample thicknesses range between 40–60 mm. By comparing samples S1, S2, and S7, the influence of wool quality on sound absorption can be observed. Sample S1 has an 80% percentage of first quality sheep wool, whereas sample S2 has a 40% percentage of first quality sheep wool and a 40% percentage of second quality sheep wool. Finally, sample S7 has an 80% percentage of second quality sheep wool. As observed from Figure 6, the quality of sheep wool does not have an influence on the absorption coefficient. Therefore, low quality sheep wool, which cannot be used in the textile industry, can be used as a sound absorbing material.

**Figure 7:**
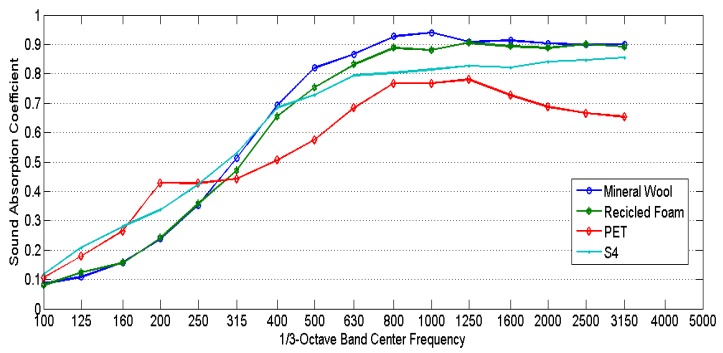
Comparison between the diffuse field sound absorption coefficients of sheep wool sample S4, mineral wool, recycled poliurethane foam and polyester fibers (PET).

The comparison between the diffuse field sound absorption coefficients of sheep wool sample S4, mineral wool, recycled poliurethane foam [27], and PET is shown in Figure 7. All samples have the same thickness of 40 mm. The bulk densities of the samples were mineral wool 30 kg/m^3^, recycled foam 80 kg/m^3^, and PET 35 kg/m^3^. The sound absorption curves have similar shapes and values, except for the PET sample. There are some differences in the low frequency range where PET and sheep wool have higher values than mineral wool and recycled foam. 

From the measured diffuse field sound absorption coefficients, and in accordance with ISO 11654 standard [26], the weighted sound absorption coefficient, *α*_w_, can be obtained for all the measured samples in the reverberation chamber. Table 4 shows the weighted sound absorption coefficient, *α*_w_. It is observed that sheep wool samples have the same or, in samples S2, S5, and S6, even higher sound absorbing capacity than mineral wool or recycled polyurethane foams.

## 5. Calculation of the Sound Absorption Coefficient of Sheep Wool

It is desirable to have a method for predicting sound absorbing performance of sheep wool due to the fact that it is impossible to measure every possible sample. In this section, a summary of the model for predicting the sound absorbing performance of sheep wool is given. This model is based on the empirical model proposed by Delany and Bazley [28]. Two complex values determine the sound propagation through a homogeneous and isotropic material in the frequency domain. They are the characteristic wave impedance (*Z*) and the characteristic propagation constant (*k'*). Delany and Bazley model determines the real constants *C_i_* (*i* = 1 … 8) that best fit the following Equations (1) and (2):

(1) $$Z=Z_{0}\left(1+C_{1}\chi^{-C_{2}}-jC_{3}\chi^{-C_{4}}\right)$$

(2) $$k\prime =k\left(C_{5}\chi^{-C_{6}}+j\left(1+C_{7}\chi^{-C_{8}}\right)\right)$$

where *Z*_0_ = *ρc* is the characteristic impedance of air, *χ* = *ρf*/*σ* is a dimensionless parameter, *ρ* is the air density at room temperature (≈1.2 kg/m^3^), *f* is the sound frequency, *σ* is the airflow resistivity (rayls/m), *c* is the speed of sound in air at room temperature (≈343 m/s), and *k* = *ω*/*c* = 2*πf*/*c* is the free field wavenumber. The range over which the model is valid is 0.012 ≤ *χ* ≤ 1.2. 

The normal-incidence sound absorption coefficient, *α*, can be determined by Equation (3) [19]:

(3) $$\alpha =\frac{4Z_{0}Z_{dR}}{{\left|Z_{d}\right|}^{2}+2Z_{0}Z_{dR}+Z_{0}^{2}}$$

where the rigid-backing specific surface impedance of the material is given by

(4) $$Z_{d}=Z_{0}\coth\left(k\prime d\right)=Z_{dR}+jZ_{dI}$$

where *d* is the material layer thickness and *Z_dR_* and *Z_dI_* are the real and imaginary parts of *Z_d_*, respectively.

The regression coefficients, *C_i_*, are determined from the normal-incidence sound absorption coefficients in the frequency domain of the measured data for a known airflow resistivity of the material sample. An iterative method based on a minimization of a quadratic error function is used to obtain the regression coefficients that best fit the measured sound absorption coefficient.

The quadratic error function used in the iterative process is defined as

(5) $$\varepsilon =\sum_{i=1}^{N}{\left(\alpha_{i}-{\hat{a}}_{i}\right)}^{2}$$

where *α_i_* is the measured normal-incidence sound absorption coefficient for a material sample at the *i*-th frequency and *â_i_* is the corresponding value estimated from Equations (1) and (2). Minimization of Equation (5) implies that

(6) $$\frac{\partial \varepsilon }{\partial C_{i}}=2\sum_{i=1}^{N}\left(\alpha_{i}-{\hat{a}}_{i}\right)\frac{\partial {\hat{a}}_{i}}{\partial C_{i}}=0$$

for all *i* = 1 … 8.

To minimize the nonlinear Equation (6) and to obtain the corresponding values of *C_i_*, a MATLAB computer program was implemented. The Nelder-Mead simplex method [29] was used for the optimization process. Acceptable values of *χ* were constrained to fall between 0.04 and 1.0. The regression coefficients of the empirical model proposed by Delany and Bazley [28] were used as initial values in the iterative method.

**Figure 8:**
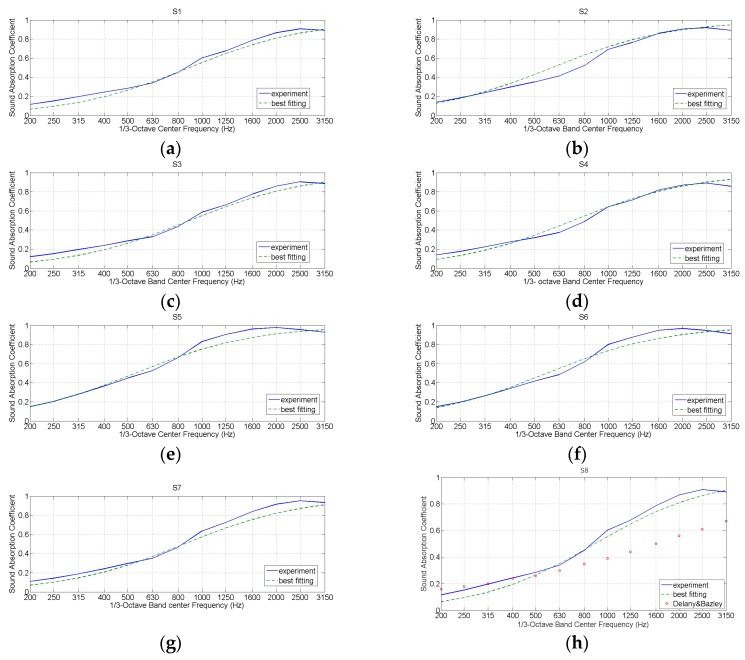
Best-fit curves for the different sheep wool samples measured in this paper. (**a**): sample S1; (**b**): sample S2; (**c**): sample S3; (**d**): sample S4; f; (**e**): sample S5; (**f**): sample S6; (**g**): sample S7 and (**h**): sample S8.

Table 5 reports the obtained coefficients, while Figure 8 shows the results of the best-fit curves normal incidence sound absorption coefficient for the sheep wool samples considered. It is interesting to emphasize that the empirical model proposed by Delany-Bazley, as it can be observed from Figure 8h, does not adjust to the absorption coefficient measurements for one of the sheep wools analyzed in this work. In the same figure, it can be observed that the model developed in this work, specific for sheep wool, adjusts much better to the experimental values. 

## 6. Conclusions

In this paper, the sound absorption performance of sheep wool has been investigated. Seven sheep wool samples with different wool compositions and densities have been used. Airflow resistance and sound absorption coefficients at normal and diffuse incidence measurements were carried out. From the measurements results, it has been demonstrated that sheep wool is a good sound absorbing material at medium and high frequencies. It is also shown that sheep wool has comparable sound absorption performance to that of mineral wool or recycles polyurethane foams. An empirical model proposed by Delany-Bazley has been used to predict the sound absorption of the sheep wool samples. A reasonable agreement for sound absorption has been obtained.

## Figures and Tables

**Table 1:** Rating of sound absorption according to ISO 11654, 1997.

Sound Absorption Rating	*α*_w_
A	0.90 or higher
B	between 0.8 and 0.85
C	between 0.6 and 0.75
D	between 0.3 and 0.55
E	between 0.15 and 0.25
No qualify	0.10 or lower

**Table 2:** Composition and the code used for each sample.

Sample	Composition						
	Pet Bi-Co	1st Quality Wool	2nd Quality Wool	Density (Kg/m^3^)	Weight (g/m^2^)	Nominal Thickness (mm)	Test Thickness (mm)
S1	20	80	0	30	1500	50	43
S2	20	40	40	30	1500	50	53
S3	20	40	40	25	1250	50	43
S4	20	40	40	30	1200	40	53
S5	20	40	40	30	1800	60	55
S6	20	40	40	40	2000	50	54
S7	20	0	80	30	1500	50	44

**Table 3:** Specific airflow resistance measurement results.

Sample	Density (Kg/m^3^)	Weight (g/m^2^)	Nominal Thickness (mm)	Test Thickness (mm)	Ingard & Dear (rayls/m) × 1000	Dragonetti (rayls/m) × 1000
S1	30	1500	50	43	9.29–9.31	6.89–7.11
S2	30	1500	50	53	7.59–7.61	5.26–5.61
S3	25	1250	50	43	9.33–9.47	6.70–6.90
S4	30	1200	40	53	9.28–9.32	6.34–6.47
S5	30	1800	60	55	7.18–7.22	5.85–6.05
S6	40	2000	50	54	7.38–7.42	7.55–7.59
S7	30	1500	50	44	9.09–9.13	7.52–7.68

**Table 4:** Measured rating of sound absorption according to ISO 11654.

Sample	*α*_w_ (11654:1958)	Absorption Class
S1	0.75	C
S2	0.80	B
S3	0.75	C
S4	0.75	C
S5	0.85	B
S6	0.80	B
S7	0.75	C
Mineral Wool	0.65	C
Recicled Foam	0.65	C
PET	0.70	C

**Table 5:** Values of coefficients C_i_.

Model	C1	C2	C3	C4	C5	C6	C7	C8
**Delany & Bazley**	0.057	0.754	0.087	0.732	0.189	0.595	0.098	0.700
**First Quality Sheep Wool**	0.0574	0.3808	0.0222	0.8552	0.1999	0.8906	0.1031	0.8687
**Second Quality Sheep Wool**	−0.0298	0.4829	0.0249	0.7419	0.2653	1.1107	0.0965	1.0131
**Sheep Wool Mixture**	0.0560	0.3640	0.0164	0.9325	0.1915	0.7863	0.1201	1.1163

## References

[1] Pinto J., Cruz D., Paiva A., Pereira S., Tavares P., Fernandes L., Varum H. (2012). Characterization of corn cob as a possible raw building material. Constr. Build. Mater..

[2] Briga-Sa A., Nascimento D., Teixeira N., Pinto J., Caldeira F., Varum H., Paiva A. (2013). Textile waste as an alternative thermal insulation building material solution. Constr. Build. Mater..

[3] Bicini H., Eken M., Dolaz M., Aksogan O., Kara M. (2014). An environmentally friendly thermal insulation material from sunflower stalk, textile waste and stubble fibres. Constr. Build. Mater..

[4] Korjenic A., Klaric S., Hadžić A., Korjenic S. (2015). Sheep Wool as a Construction Material for Energy Efficiency Improvement. Energies.

[5] Lopez-Hurtado P., Rouilly A., Vandenbossche V., Raynaud C. (2016). A review on the properties of cellulose fibre insulation. Build. Environ..

[6] Lopez-Hurtado P., Rouilly A., Raynaud C., Vandenbossche V. (2016). The properties of cellulose insulation applied via the wet spray process. Build. Environ..

[7] Binicia H., Aksoganb O., Demirhan C. (2016). Mechanical, thermal and acoustical characterizations of an insulationcomposite made of bio-based materials. Sustain. Cities Soc..

[8] Asdrubali F., Bianchi F., Cotana F., D’Alessandro F., Pertosa M., Pisello A.L., Schiavoni S. (2016). Experimental thermo-acoustic characterization of innovative common reed bio-based panels for building envelope. Build. Environ..

[9] Ballagh K.O. (1996). Acoustical Properties of Wool. Appl. Acoust..

[10] Ersoy S., Küçük H. (2009). Investigation of industrial tea-leaf-fibre waste material for its sound absorption properties. Appl. Acoust..

[11] Oldham D.J., Egan C.A., Cookson R.D. (2011). Sustainable acoustic absorbers from the biomass. Appl. Acoust..

[12] Berardi U., Iannace G. (2015). Acoustic characterization of natural fibers for sound absorption applications. Build. Environ..

[13] Mati-Baouche N., Baynast H., Michaud P., Dupont T., Leclaire P. (2016). Sound absorption properties of a sunflower composite made from crushed stem particles and from chitosan bio-binder. Appl. Acoust..

[14] Rwawiire S., Tomkova B., Militky J., Hes L., Kale B.M. (2017). Acoustic and thermal properties of a cellulose nonwoven natural fabric (barkcloth). Appl. Acoust..

[15] López J.P., El-Mansouri N.E., Alba J., Del-Rey R., Mutjé P., Vilaseca F. (2012). Acoustic properties of polypropylene composites reinforced with stone groundwood. BioResources.

[16] Arenas J.P., Rebolledo J., Del Rey R., Alba J. (2014). Sound absorption properties of unbleached cellulose loose-fill insulation material. BioResources.

[17] Reixach R., Del Rey R., Alba J., Arbat G., Espinach F.X., Mutjé P. (2015). Acoustic properties of agroforestry waste orange pruning fibers reinforced polypropylene composites as an alternative to laminated gypsum boards. Constr. Build. Mater..

[18] Del Rey R., Alba J., Ramis J., Sanchis V. (2011). New absorbent acoustics materials from plastic bottle remnants. Mater. Constr..

[19] Ramis J., Alba J., Del Rey R., Escuder E., Sanchís V. (2010). New absorbent material acoustic based on kenaf’s fibre. Mater. Constr..

[20] Baxter B.P., Cottle D.J. Fibre Diameter Distribution Characteristics of Midside (Fleece) Samples and Their Use in Sheep Breeding. Proceedings of the Boston Meeting of the International Wool Textile Organization.

[21] Ingard K.U., Dear T.A. (1985). Measurement of Acoustic Flow Resistance. J. Sound Vib..

[22] Dragonetti R., Ianniello C., Romano A.R. (2010). Measurement of the resistivity of porous materials with an alternating air-flow method. J. Acoust. Soc. Am..

[23] Del Rey R., Alba J., Arenas J.P., Ramis J. (2013). Evaluation of two alternative procedures for measuring airflow resistance of sound absorbing materials. Arch. Acoust..

[24] (1998). Acoustics-Determination of Sound Absorption Coefficient and Impedance in Impedance Tubes-Part 2: Transfer-function Method.

[25] (2003). Acoustics-Measurement of Sound Absorption in a Reverberation Room.

[26] (1997). Acoustics-Sound Absorbers for Use in Buildings-Rating of Sound Absorption.

[27] Del Rey R., Alba J., Arenas J.P., Sanchis V. (2012). An empirical modelling of porous sound absorbing materials made of recycled foam. Appl. Acoust..

[28] Delany M.E., Bazley E.N. (1970). Acoustical properties of fibrous absorbent materials. Appl. Acoust..

[29] Lindfield G., Penny J. (1995). Numerical Methods using MATLAB, Ellis Horwood Series in Mathematics & Its Applications.

